# Generation and characterization of CD19-iCre mice as a tool for efficient and specific conditional gene targeting in B cells

**DOI:** 10.1038/s41598-021-84786-6

**Published:** 2021-03-09

**Authors:** Tomoharu Yasuda, Yuichi Saito, Chisato Ono, Kazuhiko Kawata, Akemi Baba, Yoshihiro Baba

**Affiliations:** 1 Division of Immunology and Genome Biology, Medical Institute of Bioregulation, Kyushu University, 3-1-1 Maidashi, Higashi-ku, Fukuoka, 812-8582 Japan; 2 Department of Immunology, Graduate School of Biomedical and Health Sciences, Hiroshima University, Hiroshima, 739-8511 Japan

**Keywords:** Humoral immunity, B cells

## Abstract

The Cre/loxP system is a powerful tool for generating conditional gene knockout (KO) mice and elucidate gene function in vivo. CD19-Cre and Mb1-iCre transgenic mice are commonly used for generating B cell-specific KO mice and investigate the development, as well as the physiological and pathophysiological roles of B cells. However, the CD19-Cre line low efficiency and the Mb1-iCre line occasional ectopic recombination represent challenges for their use. Thus, we developed a CD19-codon-improved Cre (CD19-iCre) knock-in mouse with the T2A-iCre sequence inserted into the *Cd19* locus, just before the stop codon. The CD19-iCre mice were compared with existing models, crossed with the Rosa26-EYFP reporter mice, and their recombination activity in B cells carrying different Cre alleles was assessed. CD19-iCre mice showed more effective Cre recombination in the early B cell developmental stages compared with the CD19-Cre mice. The efficiencies of the CD19-iCre and Mb1-iCre lines were similar; however, the B lineage-specific recombination was more stringent in the CD19-iCre line. Furthermore, the utility value of the CD19-iCre model was superior than that of the CD19-Cre mice regarding deletion efficiency in IL10-floxed mice. Thus, the CD19-iCre line is a valuable tool for highly efficient gene targeting specific to the B cell compartment.

## Introduction

B cells play a vital role in humoral immunity against pathogens^1,2^. In the context of diseases such as autoimmunity, inflammation, and allergy, B cells can act as effector cells and initiate an immune response through antibody production, antigen presentation, and secretion of inflammatory cytokines^3–5^. In addition, recent evidence shows that B cells can function as negative regulators of immunity via anti-inflammatory cytokines such as interleukin (IL)-10 and IL-35^5–7^. The peripheral B cell pool is derived from hematopoietic stem/progenitor cells in the bone marrow. The development of B-lineage cells proceeds through several stages, which are orchestrated by successive steps involved in the expression and assembly of immunoglobulin genes and a complex network of transcription factors^1,8,9^.

B cell-specific conditional knockout (BKO) mouse models have opened invaluable avenues for determining the molecular mechanisms regulating the development of B cells and the pathophysiological functions in which they are involved. The current options for generating pan-BKO mice are by employing CD19-Cre^10^ or Mb1-codon-optimized improved Cre (iCre)^11^ mice, in which Cre recombinase is able to delete the regions of the genome flanked by *loxP* sites (termed as floxed). These Cre driver lines (CD19-Cre and Mb1-iCre) have been well characterized and used to successfully assess the function of genes through their deletion or genetic manipulation in a B cell-specific manner^10,11^. However, Cre-mediated deletion in the CD19-Cre and Mb1-iCre lines exhibits flaws with regard to its efficiency and specificity, respectively. When B cell-specific Cre mice were crossed with mice harboring the Rosa26-enhanced yellow fluorescent protein (EYFP) reporter (R26EYFP^+/fl^; hereafter referred to as *R26*^*EYFP*^ mice)^12^ which express EYFP directly from the ubiquitous *Rosa26* promoter following Cre-dependent deletion of a *lox*P-flanked stop element, it was possible to evaluate the specificity and efficiency of Cre expression in B cells using flow cytometry. A very small population of EYFP^+^ cells was identified among pro-B cells of CD19-Cre/Rosa26-EYFP double transgenic mice (*Cd19*^*Cre*/+^*R26*^*EYFP*^)^13^, and less than half of total CD19^+^ B cells in the bone marrow of these mice expressed EYFP, whereas CD19 expression began at the pro-B cell stage in the bone marrow during B cell development^14^. In contrast, the other B cell-specific Cre driver line Mb1-iCre, which was created by replacing the coding exons of the *Cd79a* (*Mb1*) locus with iCre recombinase-encoding cDNA, showed efficient *Cre* deletion in bone marrow B cells, including in pro-B cells, when crossed with *R26*^*EYFP*^ mice^11,13^. Thus, researchers often use the Mb1-iCre line for the deletion or manipulation of a gene to analyze early B cell development in the bone marrow.

With respect to peripheral tissues such as the spleen and lymph nodes, both *Cd19*^*Cre*/+^*R26*^*EYFP*^ and Mb1-iCre/Rosa26-EYFP (*Mb1*^*iCre/*+^*R26*^*EYFP*^) lines targeted B cells with high frequency (approximately 80–93% and ~ 99% of B cells, respectively)^11,13^. Therefore, these data indicate that Mb1-iCre mice are more efficient than CD19-Cre mice in the recombination of *lox*P sites during B cell development. However, it should be noted that after crossing with *R26*^*EYFP*^ reporter mice, there can be a small fraction of EYFP^+^ T cells detected in the Mb1-iCre line, suggesting minor aberrant *lox*P recombination^11^. In addition, several studies reported the occurrence of Mb1-iCre-mediated recombination in the germline in some cases^15–18^. Although deletion efficiency using the same Cre driver can also vary depending on the floxed alleles, such unexpected Cre expression in the germline can lead to gene deletions being passed to the subsequent generation. Therefore, breeding strategies should be carefully adjusted for the maintenance of these lines^19^.

To overcome these limitations of the currently available B cell specific Cre lines, we developed a new B-lineage Cre mouse model, which is a *Cd19-T2A-iCre* knock-in line (CD19-iCre), in which the *T2A-iCre* sequence is inserted in-frame to the 3′ end of the *Cd19* coding sequence (immediately upstream of the stop codon). By crossing with the *R26*^*EYFP*^ line, we show that the CD19-iCre driver line enables highly efficient and specific ablation of floxed genes in the B cell lineage from the pro-B cell stage without infidelity of Cre-mediated recombination in T cells. When crossed with IL10-floxed mice, the CD19-iCre line was found to be superior to CD19-Cre mice with regard to the disruption of IL-10 production in B cells. Thus, the CD19-iCre line provides a new option for generating B cell-specific deficient mice with high specificity and efficiency, which further facilitates investigating the functional roles of a gene throughout B lineage cells.

## Results

### Generation of the CD19-iCre knock-in mice

**Figure 1:**
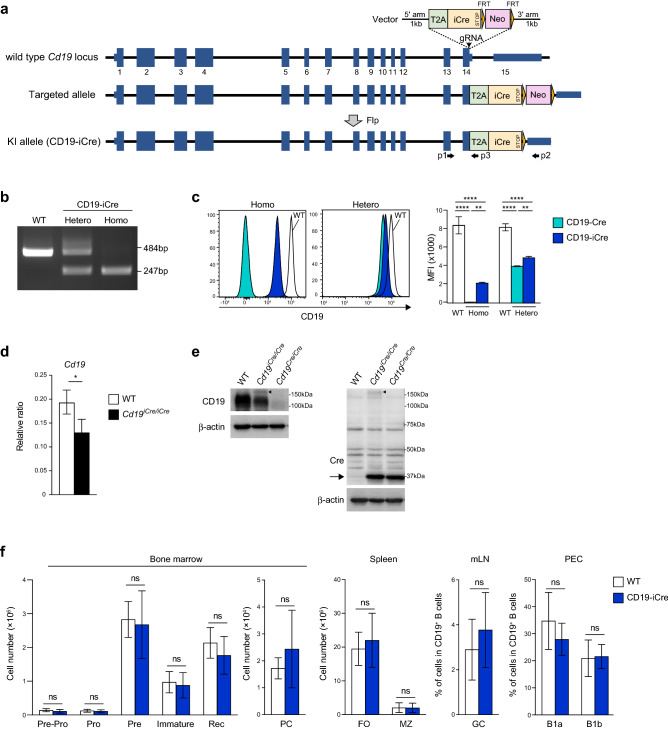
Generation of the CD19-iCre knock-in mice. (**a**) Schematic representation of the targeting strategy. The sequences for the T2A-iCre have been inserted between the last amino acid and the stop codon in exon 14 of *Cd19* locus. Neomycin resistance gene cassette (Neo) is flanked by *Frt* sites and inserted downstream of the untranslated region. The KI allele is obtained after Flippase (Flp) site-directed recombination of the selection marker. (**b**) PCR for detection of *iCre* transgene KI allele with genomic DNA from wild-type (WT), heterozygous and homozygous CD19-iCre mice. Amplicons of 484 and 247 bp are identified by primer pairs (p1–p2 and p1–p3) specific for the WT and iCre alleles, respectively. (**c**) Flow cytometry of the surface expression of CD19 on B220^hi^ B cells of peripheral blood from WT, heterozygous and homozygous CD19-Cre and CD19-iCre mice. Right, mean fluorescence intensity (MFI) of the staining of CD19. (**d**) Quantitative RT-PCR of mRNA encoding CD19 in WT and CD19^*iCre*/*iCre*^ B cells, normalized to the expression of β-actin. (**e**) Western blotting analysis of whole-cell lysates of splenic B cells from WT, CD19^*iCre*/*iCre*^, and CD19^*Cre*/*Cre*^ mice with antibodies specific for CD19, Cre, and β-actin. The arrowheads and arrow indicate fusion proteins (CD19-T2A-iCre) and Cre/iCre proteins, respectively. (**f**) Absolute number or frequency of each B cell subset from bone marrow, spleen, mesenteric lymph node (mLN) and peritoneal cavity (PEC) and from WT and CD19^*iCre*/+^ mice on the basis of total cell count and flow cytometry analysis. B cell subsets were as follow: pre-pro-B (IgM^−^IgD^−^B220^+^CD19^−^CD43^+^), pro-B (IgM^−^IgD^−^B220^+^CD19^+^CD43^+^), pre-B (IgM^−^IgD^−^B220^+^CD19^+^CD43^−^), immature B (IgM^+^IgD^-^B220^low^CD19^+^), recirculating B (Rec: IgM^+^IgD^+^B220^hi^CD19^+^) and plasma cells (PC; CD138^+^TACI^+^); follicular (FO; CD19^+^CD93^-^CD21^low^CD23^hi^), marginal zone (MZ; CD19^+^CD93^−^CD21^hi^CD23^low^); B1a (IgM^+^CD5^+^CD43^+^B220^low^CD19^+^), B1b (IgM^+^CD5^−^CD43^+^B220^low^CD19^+^); germinal center B cells (GC; CD19^+^Fas^+^CD38^−^). Data are representative of three independent experiments (**b**, **d**, **e**), or pooled from three (**c**) or two (**f**) independent experiments. Data are presented as mean ± SD. *P < 0.05; **P < 0.01; ***P < 0.001; ****P < 0.0001; ns, not significant. The *P* values were obtained by one-way ANOVA with Tukey’s post hoc test (**c**) or two-tailed unpaired t test (**d**, **f**).

To generate a mouse line that expresses the iCre recombinase^20^ in CD19-expressing B lineage cells without disturbing its endogenous *Cd19* expression, we generated a CD19-T2A-iCre knock-in line, in which the T2A-iCre sequence was inserted in-frame to the 3′-end of the *Cd19* coding sequence (immediately upstream of the stop codon) (Fig. 1a). Following the induction of double-strand DNA breaks by the Cas9 enzyme mediated by a guide RNA (gRNA), the homology arms guided the T2A-iCre to be inserted in-frame with the *Cd19* open reading frame by homologous directed repair. This targeting strategy using self-cleaving 2A sequences^21^ was designed to mediate bicistronic translation, which is potentially more efficient than the well-described internal ribosome entry site sequence. Upon translation of the chimeric *CD19-T2A-iCre* mRNA, the *T2A* sequence led to ribosome skipping^22^, resulting in the co-expression of CD19 and iCre as discrete proteins in CD19-expressing cells. Consequently, iCre expression was regulated by the *Cd19* promoter in tandem with the endogenous CD19 expression. In the present study, the targeting construct was correctly introduced in embryonic stem (ES) cells, which was confirmed by polymerase chain reaction (PCR) analysis. This was followed by deletion of a neomycin resistance gene (*Neo*^R^) cassette (Neo cassette) by *Flp* cDNA transfection into targeted ES cells to avoid unpredictable Cre activity^23^. After confirming the Neo cassette deletion by PCR, the targeted ES cells were transferred into blastocysts and the mouse germline (Fig. 1b). Heterozygous and homozygous CD19-iCre mice (referred to as *Cd19*^*iCre/*+^ and *Cd19*^*iCre/iCre*^, respectively) were found to be viable, fertile, and born at the expected Mendelian frequencies (data not shown).

To understand the consequences of the CD19-iCre targeted insertion strategy on CD19 expression, we analyzed peripheral B cells in mice heterozygous or homozygous for the knock-in allele by flow cytometry and compared the collected data with that of a previously published CD19-Cre line^10^, in which the Cre was inserted in exon 2 and the CD19 coding sequence was disrupted, leading to a CD19 deficiency in the homozygous situation. CD19-Cre homozygous mice showed complete loss of CD19 expression, whereas the CD19-iCre homozygous mice retained CD19 expression, albeit there was a significant reduction in its levels compared with that in B cells from wild-type (WT) mice. The mean fluorescence intensity (MFI) of CD19-stained B cells in CD19-iCre mice decreased by about fourfold compared with WT mice (Fig. 1c). Moreover, Cd19 mRNA and protein levels were also reduced in splenic B cells from *Cd19*^*iCre/iCre*^ mice (Fig. 1d, e), as assessed by quantitative RT-PCR and western blot analysis, respectively. It should be noted that high levels of released iCre and CD19 along with subtle levels of the uncleaved CD19-T2A-iCre fusion protein were detected, indicating efficient T2A self-processing in B cells (Fig. 1e). These results suggest that the targeted insertion of T2A-iCre might interfere with *Cd19* expression or mRNA stability. Heterozygous mice for the Cre allele are commonly used as Cre deleter lines. Mice heterozygous for the Cre insertion in CD19-Cre mice, which retained one functional CD19 allele, showed reduced surface CD19 levels (the MFI was approximately half of that of WT mice) (Fig. 1c). *Cd19*^*iCre/*+^ mice also showed reduced CD19 expression, but significantly higher compared with CD19-Cre mice (Fig. 1c). However, B cells from the *Cd19*^*iCre/*+^ mice did not show any further apparent alterations and exhibited normal development in the bone marrow, spleen, lymph nodes, and peritoneal cavity (Fig. 1f).

### Efficient recombination at different B cell developmental stages with the CD19-iCre transgene

**Figure 2:**
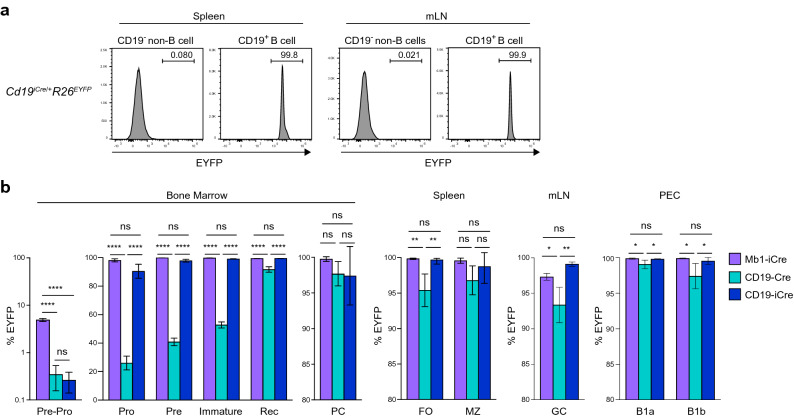
B cell-specific and efficient Cre-mediated recombination activity in CD19-iCre line. (**a**) EYFP expression is demonstrated in CD19^+^ B cells and CD19^-^ non-B cells from spleen and mLN of *Cd19*^*iCre*/+^*R26*^*EYFP*^ mice. (**b**) Flow cytometry analysis of EYFP expression by different B cell subsets (as defined in Fig. 1f), from bone marrow, spleen, peritoneal cavity (PEC), and mesenteric lymph node (mLN) of *Mb1*^*iCre*/+^*R26*^*EYFP*^*, Cd19*^*Cre*/+^*R26*^*EYFP*^, and *Cd19*^*iCre*/+^*R26*^*EYFP*^ mice. Data are representative of three independent experiments (**a**), or pooled from two independent experiments (**b**). Data are presented as mean ± SD. *P < 0.05; **P < 0.01; ***P < 0.001; ****P < 0.0001; ns, not significant. The *P* values were obtained by one-way ANOVA with Tukey’s post hoc test (**b**).

To determine the efficiency of Cre expression, CD19-iCre mice were crossed with the *R26*^*EYFP*^ line to generate *Cd19*^*iCre/*+^*R26*^*EYFP*^ mice. Almost all CD19^+^ B cells expressed EYFP in the spleen and mesenteric lymph nodes (mLN), but EYFP was not detected in CD19^−^ non-B cells (Fig. 2a), suggesting high B cell-specific Cre expression in the CD19-iCre lines. Next, we examined the efficiency of Cre-mediated deletion in B cell subsets of CD19-iCre mice and compared them side-by-side with previously published B cell specific Cre-driver lines: the CD19-Cre and the Mb1-iCre mice that were also crossed with the *R26*^*EYFP*^ mice. A previous study demonstrated that Mb1-iCre mice have an earlier and/or more efficient *lox*P site recombination in developing B cells than CD19-Cre mice^11^. Our results also confirmed these findings, since almost all of the pro-B cells expressed EYFP in *Mb1*^*iCre/*+^*R26*^*EYFP*^ mice, whereas only ∼50% of the same subset of cells were EYFP-positive in *Cd19*^*Cre/*+^*R26*^*EYFP*^ mice (Fig. 2b).

A comparison of our newly developed CD19-iCre line showed highly efficient Cre-mediated activation of EYFP expression in pro-B cells, similar to that in the Mb1-iCre line. In the case of the very early B lineage stage (pre-pro-B cell stage), before the expression of CD19, EYFP^+^ cells were observed only in *Mb1*^*iCre/*+^*R26*^*EYFP*^ mice. Regarding the peripheral tissues, splenic follicular B cells, germinal center B cells, and B1a/B1b cells in the peritoneal cavity of *Cd19*^*iCre/*+^*R26*^*EYFP*^ mice exhibited higher efficiency than that in *Cd19*^*Cre/*+^*R26*^*EYFP*^ mice, but equivalent to that in *Mb1*^*iCre/*+^*R26*^*EYFP*^ mice (Fig. 2b). Other B cell subsets in the spleen (marginal zone B cells) and bone marrow (plasma cells and recirculating B cells) were comparable among the three Cre lines (Fig. 2b). These results demonstrate the highly efficient Cre-mediated recombination in B lineage cells from the pro-B cell stage in CD19-iCre mice.

### Highly specific recombination in B cells with the CD19-iCre transgene

**Figure 3:**
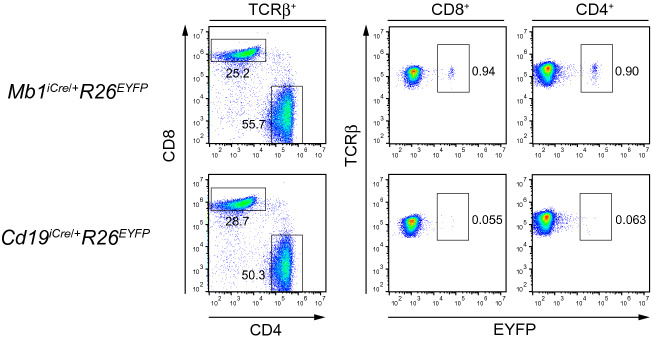
Cre-mediated recombination in T cells is not observed in CD19-iCre/Rosa26-EYFP double transgenic mice. EYFP expression is demonstrated in splenic TCRβ^+^CD4^+^ and TCRβ^+^CD8^+^ T cells of *Mb1*^*iCre*/+^*R26*^*EYFP*^ and *Cd19*^*iCre*/+^*R26*^*EYFP*^ mice. Data are representative of two independent experiments.

A previous study indicated aberrant *lox*P recombination in T cells from *Mb1*^*iCre/*+^*R26*^*EYFP*^ mice, albeit with a relatively low frequency^11^. To detect ectopic Cre-mediated activation of EYFP expression in *Cd19*^*iCre/*+^*R26*^*EYFP*^ and *Mb1*^*iCre/*+^*R26*^*EYFP*^ mice, flow cytometry analysis was conducted. In agreement with a previous report^11^, a small number of splenic CD4^+^ and CD8^+^ TCRβ^+^ T cells from *Mb1*^*iCre/*+^*R26*^*EYFP*^ mice were found to be EYFP^+^ (Fig. 3). In contrast, EYFP expression was virtually absent in T cells from *Cd19*^*iCre/*+^*R26*^*EYFP*^ mice (Fig. 3). Together, these data indicate the highly targeted expression of iCre in CD19-iCre mice, thereby demonstrating that this transgenic line is a very valuable resource to study B cell-specific gene functions.

### CD19-iCre mice are a valuable line for studying B cell function

**Figure 4:**
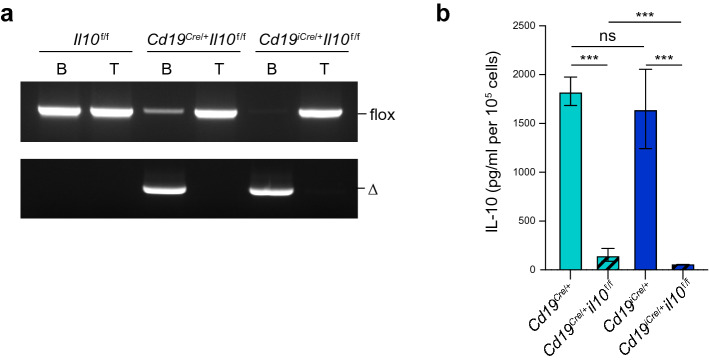
Efficient excitation of floxed allele in CD19-iCre mice. (**a**) Genomic PCR of B and T cells sorted from the spleen of *Il10*^f/f^, *Cd19*^*Cre*/+^*Il10*^f/f^, and *Cd19*^*iCre*/+^*Il10*^f/f^ mice. Amplicons are identified as floxed (flox) and Delta (Δ) alleles. (**b**) ELISA of IL-10 by B cells isolated from *Cd19*^*Cre*/+^, *Cd19*^*Cre*/+^*Il10*^f/f^, *Cd19*^*iCre*/+^, and *Cd19*^*iCre*/+^*Il10*^f/f^ mice and cultured with LPS for 48 h followed by stimulation with anti-IgM for 24 h. Data are representative of at least three independent experiments (**a**), or pooled from three independent experiments (**b**). Data are presented as mean ± SD. *P < 0.05; **P < 0.01; ***P < 0.001; ****P < 0.0001; ns, not significant. The *P* values were obtained by Mann–Whitney U test (**b**).

We have previously shown that lipopolysaccharide (LPS)-activated B cells produce IL-10 after stimulation with B cell antigen receptor (BCR)^24,25^. To test the utility of the CD19-iCre driver line for general knockout studies, this line was crossed with IL10 floxed (*Il10*^f/f^) mice^26^ to generate *Cd19*^*iCre/*+^*Il10*^f/f^ animals. We compared these *Cd19*^*iCre/*+^*Il10*^f/f^ mice with CD19-Cre/IL10^f/f^ (*Cd19*^*Cre/*+^*Il10*^f/f^) mice regarding the recombination efficiency of floxed alleles in splenic B cells. The deleted *Il10* floxed alleles in B cells, but not T cells, sorted from spleens with high purity (> 98%) were confirmed by genomic PCR in *Cd19*^*Cre/*+^*Il10*^f/f^ and *Cd19*^*iCre/*+^*Il10*^f/f^ mice (Fig. 4a). However, it is noteworthy that the recombination efficiency in B cells from *Cd19*^*iCre/*+^*Il10* ^f/f^ mice was superior to that in *Cd19*^*Cre/*+^*Il10*^f/f^ mice. Enzyme-linked immunosorbent assay (ELISA) showed that *Cd19*^*Cre/*+^*Il10*^f/f^ B cells had compromised IL-10 secretion compared with control *Cd19*^*Cre/*+^ B cells (Fig. 4b), in agreement with a previous study^27^. Notably, IL-10 production was significantly reduced in *Cd19*^*iCre/*+^*Il10*^f/f^ B cells than in *Cd19*^*Cre/*+^*Il10*^f/f^ B cells (Fig. 4b). Collectively, these data suggest that CD19-iCre mice are valuable Cre drivers to manipulate genes in B cells.

## Discussion

In this study, we describe a new B cell-specific Cre line, named CD19-iCre, which is the third-generation line after CD19-Cre and Mb1-iCre. Our findings indicate that CD19-iCre can recombine *loxP* sites with high efficiency in pro-B cells of *Cd19*^*iCre/*+^*R26*^*EYFP*^ mice and show efficient Cre-mediated deletion of *loxP* sites during B cell development in several tissues, which is comparable to that of the Mb1-iCre line but greater than that of the CD19-Cre mice. Indeed, the *Il10* floxed allele was more efficiently deleted in splenic B cells from *Cd19*^*iCre/*+^*Il10*^f/f^ mice compared with those from *Cd19*^*Cre/*+^*Il10*^f/f^ mice, and, consequently, *Cd19*^*iCre/*+^*Il10*^f/f^ B cells expressed less IL-10 than *Cd19*^*Cre/*+^*Il10*^f/f^ B cells.

Despite the previously reported studies on Mb1-iCre mice attributing the efficiency of the Cre deletion to promoter-specific differences between the Mb1-iCre and the CD19-Cre mouse lines, our present study shows that this may not be the case as the results exhibited highly efficient Cre-mediated recombination in pro-B cells of *Cd19*^*iCre/*+^*R26*^*EYFP*^ mice, which was comparable to that of *Mb1*^*iCre/*+^*R26*^*EYFP*^ mice. Given the endogenous expression of CD19 in the pro-B cell population, these results suggest that CD19-iCre mice can excise the floxed sequence more effectively than CD19-Cre mice. The enhanced efficiency of the CD19-iCre mice may be due to the use of the improved iCre, which has been reported to be more efficiently expressed in mouse cells^20^. Another possible underlying reason can be the difference in the design strategy of the Cre-transgenic lines. In the CD19-Cre line, in addition to the regulatory elements of the endogenous CD19 locus, a β-globin polyadenylation site was introduced to permit polyadenylation of the *Cre* mRNA. In the CD19-iCre line, we introduced the T2A-iCre sequence before the 3′-stop codon, with all other transcription processes of the *Cre* mRNA being retained, which might affect Cre expression in early B cell stage. Since the Cd19-Cre line used in this study possesses the Neo cassette, the recombination efficiency of the CD19-Cre mouse may be low; however, this is somewhat unlikely since it has been previously reported that excision of the *Frt*-flanked Neo cassette from the CD19-Cre allele leads to reduced Cre expression by an unknown mechanism^28^.

Conditional gene targeting based on the Cre/*lox*P system is a powerful technology for the analysis of gene function, but it is key that the expression of Cre transgenes occurs in a targeted cell-specific manner. Despite the differential sensitivity of individual *loxP*-flanked target gene alleles to Cre-mediated recombination, at least in the *R26*^*EYFP*^ reporter system, Mb1-iCre mice can exhibit undesired recombination in T cells. By contrast, our CD19-iCre mouse line did not show such ectopic recombination. Nonetheless, the proportion of detectable EYFP^+^ T cells was minor in *Mb1*^*iCre/*+^*R26*^*EYFP*^ mice, in cases in which the target genes that are to be deleted and analyzed in B cells are critical for T cell development and function. Thus, it is important to carefully assess the recombination in T cells and interpret the phenotype. Until now we did not observe Cre-mediated recombination in the germline of *Cd19*^*iCre/*+^*R26*^*EYFP*^ mice; however, we cannot exclude the possibility that the CD19-iCre line induce germline deletions.

The T2A-based approach in CD19-iCre mice may produce the equimolar amounts of iCre relative to CD19 protein from a single multicistronic transcript. Generally, a 2A peptide, when placed between genes can induce ribosomal skipping during protein translation, thereby producing discrete proteins, whereas unsuccessful skipping and continued translation may result in a fusion protein. We could detect very little CD19-T2A-iCre fusion protein in *Cd19*^*iCre/iCre*^ B cells, which might explain the observed reduction in surface CD19 levels in CD19-iCre mice. However, given the reduced *Cd19* expression in *Cd19*^*iCre/iCre*^ B cells, it seems most likely that T2A insertion may affect the expression or mRNA stability of *Cd19*. Although minor reduction in CD19 expression did not induced apparent alterations of B cell development in *Cd19*^*iCre/*+^ mice, we advocate using *Cd19*^*iCre/*+^ mice as controls for conditional BKO mice generated with this strain, as is the case with CD19-Cre or Mb1-iCre lines.

In summary, our newly developed CD19-iCre mice showed more effective Cre recombination at the early B cell stages, as well as throughout B cell development, when compared with that of the commonly used CD19-Cre line. Although the efficiency of the CD19-iCre line was comparable to that of the Mb1-iCre line, more stringent B lineage-specific recombination was observed in the novel model. Thus, its high efficiency and specificity make the CD19-iCre line the most reliable and robust model available for gene targeting and manipulation of B cells in vivo.

## Methods

### Mice

C57BL/6 mice were purchased from CLEA Japan. CD19-Cre^10^ and Rosa26-EYFP^12^ were obtained from Jackson Laboratories. The *Mb1*^*icre*/+^ (kindly provided by Dr. Michael Reth)^11^ and IL10-floxed mice (kindly provided by Dr. Werner Muller)^26^ were housed and bred under specific pathogen-free conditions in the animal facility of Kyushu University and used at the age of 7 to 15 weeks. All studies and procedures were approved by the Kyushu University Animal Experiment Committee. All animal experiments were conducted in accordance with the ARRIVE guidelines and the ethical guidelines of Kyushu University.

### Generation of the CD19-iCre knock-in mice

For the targeting vector construction, the 5′ homology arm containing the intron and exon 14 of *Cd19* (1.0 kb) and 3′ homology arm containing the 3′ UTR of *Cd19* (1.0 kb) were amplified by PCR from C57BL/6 mouse genome. Gene encoding Cre recombinase optimized for mammalian codon usage and nuclear localization signal (iCre) was obtained from Mb1-iCre mouse genome^11,20^. T2A self-cleaving peptide sequence was synthesized as oligo nucleotides adding to PCR primer to amplify iCre gene. The neomycin resistance gene (*Neo*^R^) cassette (Neo cassette) under the *tk* promoter flanked by *Frt* site sequences for Flippase (Flp) site-directed recombination was obtained from Rosa26-BCL6 targeting vector (Yasuda and Rajewsky, unpublished). Four PCR fragments were assembled in pBlueScript II plasmid vector using NEBuilder HiFi DNA assembly master mix (NEB) to generate targeting vector (pCD19_T2A_iCre Neo). The targeting vector was designed for integration of the T2A-iCre sequence just before the stop codon of *Cd19* to produce CD19 and iCre from same transcript. The Neo cassette was inserted downstream of iCre, right after the stop codon. All PCR amplifications were performed using high fidelity KOD DNA polymerase (TOYOBO). 1 × 10^6^ C57BL/6 N ES cells (EGR-101)(kindly provided by Dr. Masahito Ikawa)^29^ were electroporated with 100 ng/µl (0.61 µM) recombinant Cas9 Nuclease 3NLS (IDT #1074181), 36 ng/µl (1 µM) gCD19-1 crRNA (5′-CTAGTCACTTGGGAGTCACG//TGG-3′, stop codon is underlined):tracrRNA duplex, and 100 ng/µl pCD19_T2A_iCre Neo in 100 µl of 1xOpti-MEM medium (ThermoFisher) using NEPA 21 super electroporator (NEPA GENE) and 2 mm gap cuvette. The following parameters were used for electroporation; Poring pulse (voltage 125 V; pulse length 2.5 ms; pulse interval 50 ms; number of pulses 2; decay rate 10%; polarity +) and Transfer pulse (voltage 20 V; pulse length 50 ms; pulse interval 50 ms; number of pulses 5; decay rate 40%; polarity ±). The electrical resistivity was typically 0.03 to 0.05 kΩ. Electroporated cells were selected for 6–7 days under ES medium (StemSure D-MEM, 15% FCS, L-glutamine, non-essential amino acids, β-mercaptoethanol, and LIF) containing 200 µg/ml G418 (Nacalai tesque). 60 ES cell colonies were subjected to PCR screening, and 30 clones (50%) showed correct recombination in both 5′ and 3′ side. A clone, A4, was subjected to pCAG-Flpe electroporation (Addgene) to delete Neo cassette. *Neo*^*R*^ deleted 5–10 ES cells were injected into Balb/c blastocysts. A chimeric male (80–90% chimerism) was crossed to C57BL/6 females to obtain germline transmitted offspring.

### Antibodies

For flow cytometry, single-cell suspensions prepared from bone marrow, spleen, LNs and peripheral blood were stained with the following biotin- or fluorochrome-conjugated antibodies purchased from BioLegend, BD Biosciences or eBioscience: Biotin-conjugated anti-CD19 (1D3), and anti-CD93 (AA4.1); allophycocyanin (APC)-conjugated anti-CD5 (53-7.3), anti-CD19 (6D5), anti-CD138 (281-2), anti-TCRβ (H57-597) and anti-IgM (RMM-1); allophycocyanin (APC)-Cy7-conjugated anti-B220 (RA3-6B2), anti-CD19 (6D5); Brilliant Violet (BV450)-conjugated Streptavidin; Brilliant Violet (BV605)-conjugated anti-CD19 (6D5); phycoerythrin (PE)-conjugated anti-CD8a (53-5.7), anti-CD23 (B3B4), anti-CD95 (Jo2), anti-IgM (RMM-1), anti-TACI (8F10), and anti-TCRβ (H57-597); Phycoerythrin-Cyanine7 (PE-Cy7)-conjugated anti-CD4 (GK1.5), anti-CD21/CD35 (7E9), anti-CD38 (90), anti-CD43 (S7), and anti-CD44 (IM7); peridinin chlorophyll protein complex-cyanine 5.5 (PerCP-Cy5.5)-conjugated anti-IgD (11-26c.2a).

### Flow cytometry and cell sorting

Tissues were disrupted by passing through a 40-µm cell strainer (BD Biosciences). After red blood cell lysis with ammonium chloride potassium buffer, cells were incubated with an anti-CD16/CD32 (2.4G2; BD Pharmingen) to reduce nonspecific labeling of the cells before staining. Single cells were stained with fluorophore- or biotin-labeled antibodies. Data were acquired on a Cytoflex (Beckman Coulter) and analyzed with FlowJo software (Tree Star) to detect the following populations: pre-pro-B (IgM^−^IgD^−^B220^+^CD19^-^CD43^+^), pro-B (IgM^−^IgD^−^B220^+^CD19^+^CD43^+^), pre-B (IgM^−^IgD^−^B220^+^CD19^+^CD43^−^), immature B (IgM^+^IgD^−^B220^low^CD19^+^), recirculating B (Rec; IgM^+^IgD^+^B220^hi^CD19^+^) and plasma cells (CD138^+^TACI^+^) in the bone marrow; follicular (FO; CD19^+^CD93^-^CD21^low^CD23^hi^), and marginal zone (MZ; CD19^+^CD93^−^CD21^hi^CD23^low^) in spleen; B1a (IgM^+^CD5^+^CD43^+^B220^low^), and B1b (IgM^+^CD5^−^CD43^+^B220^low^) in peritoneal cavity (PEC); germinal center B cells (CD19^+^Fas^+^CD38^−^) in mesenteric lymph node (mLN). Cell sorting was performed on a FACSMelody (BD Biosciences) to isolate the following populations: B cells (CD19^+^TCRβ^−^) and T cells (TCRβ^+^CD19^−^) in the spleen.

### Genomic PCR analysis

Genomic DNA isolated from sorted cells or tail biopsies was used for PCR analysis. For CD19-iCre detection, the following primer pair were used: sense primer 5′-TGAAGAAGGTGGGTGTTTCTGCACTGTTTCTCGTTG-3′ and antisense primer 5′-TAGCAGACTTCCTCTGCCCTCTCCGCTTCC-3′ (*T2A-iCre*); sense primer 5′-TGAAGAAGGTGGGTGTTTCTGCACTGTTTCTCGTTG-3′ and antisense primer 5′- ATCAAGGTCATTTCACTGACTGACACCATCTGGGATC-3′ (wild-type). For *Il10*-floxed gene detection, the following primer pair were used: sense primer 5′-CCAGCATAGAGAGCTTGCATTACA-3′ and antisense primer 5′-GAGTCGGTTAGCAGTATGTTGTCCAG-3′ (*Il10*-floxed); sense primer 5′-GTGCGCCAAACACTTCTCACAGACTTGAGC-3′ and antisense primer 5′-GAGTCGGTTAGCAGTATGTTGTCCAG-3′ (*Il10*-Δ).

### Quantitative RT-PCR analysis

RNA was isolated and purified using the RNeasy kit (Qiagen) from purified B cells. cDNA was generated using the ReverTra Ace qPCR RT Master Mix (TOYOBO). Real-time PCR was performed on a LightCycler 96 (Roche) using Thunderbird SYBR qPCR mix (TOYOBO). The following primer pairs were used: *Cd19*, sense 5′-GGAGTGTCCCTGGGTCCTAT-3′ and antisense 5′-CATCCTGGGAAACCTGGTCC-3′: β-*actin*, sense 5′- GCTCTTTTCCAGCCTTC-3′ and antisense 5′-CGGATGTCAACGTCACA-3′.

### B cell isolation, stimulation and enzyme-linked immunosorbent assay

For B cell isolation, splenic B cells were purified by the negative selection of CD43^+^ cells with anti-CD43 magnetic beads (Militenyi Biotec). The purified B cell population was > 95% positive for B220 staining. For B cell stimulation assays, purified B cells (5 × 10^5^ cells/ml) were cultured in RPMI 1640 medium supplemented with 10% (vol/vol) FCS, β-mercaptoethanol and antibiotics for 48 h with 10 µg/ml of lipopolysaccharide (LPS) (Sigma-Aldrich), and then stimulated with 10 µg/ml of anti-IgM F(ab)’_2_ (Jackson Immunoresearch). IL-10 in the culture medium was detected by enzyme-linked immunosorbent (ELISA) assay according to the manufacturer’s protocol (Biolegend).

### Western blotting

Purified B cells were lysed in lysis buffer (10 mM Tris–HCl [pH 7.4], 150 mM NaCl, 1% [vol/vol] Triton X-100, 0.5 mM EDTA) plus protease inhibitor cocktails (Nacalai Tesque). Samples were transferred to polyvinyldifluoride membranes by electrophoresis and antibodies against the following proteins were used for immunodetection: antibodies against CD19 (C1C3), Cre (Poly9080), and β-actin (C-11) was obtained from GeneTex, BioLegend, and Santa Cruz Biotechnology, respectively.

### Statistical analysis

GraphPad Prism 6 (GraphPad Software) was used for all statistical analyses. Statistical significance was determined by two-tailed paired or unpaired Student’s t test for two groups or one-way ANOVA with Tukey’s post hoc test for multiple groups. Comparisons of two nonparametric data sets were done by the Mann–Whitney U test. A *p* value of less than 0.05 was considered statistically significant.

## Supplementary Information

Supplementary Information.

## References

[1] Kurosaki T, Shinohara H, Baba Y (2010). B cell signaling and fate decision. Annu. Rev. Immunol..

[2] Cyster JG, Allen CDC (2019). B cell responses: cell interaction dynamics and decisions. Cell.

[3] Sanz I, Lee FE-H (2010). B cells as therapeutic targets in SLE. Nat. Rev. Rheumatol..

[4] Rawlings DJ, Metzler G, Wray-Dutra M, Jackson SW (2017). Altered B cell signalling in autoimmunity. Nat. Rev. Immunol..

[5] Shen P, Fillatreau S (2015). Antibody-independent functions of B cells: a focus on cytokines. Nat. Rev. Immunol..

[6] Baba Y, Matsumoto M, Kurosaki T (2015). Signals controlling the development and activity of regulatory B-lineage cells. Int. Immunol..

[7] Baba Y, Saito Y, Kotetsu Y (2020). Heterogeneous subsets of B-lineage regulatory cells (Breg cells). Int. Immunol..

[8] Hardy RR, Kincade PW, Dorshkind K (2007). The protean nature of cells in the B lymphocyte lineage. Immunity.

[9] Matthias P, Rolink AG (2005). Transcriptional networks in developing and mature B cells. Nat. Rev. Immunol..

[10] Rickert RC, Roes J, Rajewsky K (1997). B lymphocyte-specific, Cre-mediated mutagenesis in mice. Nucl. Acids Res..

[11] Hobeika E (2006). Testing gene function early in the B cell lineage in mb1-cre mice. Proc. Natl. Acad. Sci. USA.

[12] Srinivas S (2001). Cre reporter strains produced by targeted insertion of EYFP and ECFP into the ROSA26 locus. BMC Dev. Biol..

[13] Audzevich T (2017). Pre/pro-B cells generate macrophage populations during homeostasis and inflammation. Proc. Natl. Acad. Sci. USA.

[14] Demircik F, Buch T, Waisman A (2013). Efficient B cell depletion via diphtheria toxin in CD19-Cre/iDTR mice. PLoS ONE.

[15] Schmidt-Supprian M, Rajewsky K (2007). Vagaries of conditional gene targeting. Nat. Immunol..

[16] Rowh MA (2011). Tp53 deletion in B lineage cells predisposes mice to lymphomas with oncogenic translocations. Oncogene.

[17] DeMicco A (2015). B cell-intrinsic expression of the HuR RNA-binding protein is required for the T cell-dependent immune response in vivo. J. Immunol..

[18] Liu R (2018). Proapoptotic BIM impacts B lymphoid homeostasis by limiting the survival of mature B cells in a cell-autonomous manner. Front. Immunol..

[19] Song AJ, Palmiter RD (2018). Detecting and avoiding problems when using the Cre-lox system. Trends Genet..

[20] Shimshek DR (2002). Codon-improved Cre recombinase (iCre) expression in the mouse. Genesis.

[21] Szymczak AL (2004). Correction of multi-gene deficiency in vivo using a single 'self-cleaving' 2A peptide-based retroviral vector. Nat. Biotechnol..

[22] Donnelly MLL (2001). Analysis of the aphthovirus 2A/2B polyprotein 'cleavage' mechanism indicates not a proteolytic reaction, but a novel translational effect: a putative ribosomal 'skip'. J. Gen. Virol..

[23] Pham CT, MacIvor DM, Hug BA, Heusel JW, Ley TJ (1996). Long-range disruption of gene expression by a selectable marker cassette. Proc. Natl. Acad. Sci. USA.

[24] Matsumoto M (2011). The calcium sensors STIM1 and STIM2 control B cell regulatory function through interleukin-10 production. Immunity.

[25] Matsumoto M (2014). Interleukin-10-producing plasmablasts exert regulatory function in autoimmune inflammation. Immunity.

[26] Roers A (2004). T cell-specific inactivation of the interleukin 10 gene in mice results in enhanced T cell responses but normal innate responses to lipopolysaccharide or skin irritation. J. Exp. Med..

[27] Teichmann LLL (2012). B cell-derived IL-10 does not regulate spontaneous systemic autoimmunity in MRL.Fas(lpr) mice. J. Immunol..

[28] Schmidt-Supprian M, Wunderlich FT, Rajewsky K (2007). Excision of the Frt-flanked neo R cassette from the CD19cre knock-in transgene reduces Cre-mediated recombination. Transgenic Res..

[29] Fujihara Y, Kaseda K, Inoue N, Ikawa M, Okabe M (2013). Production of mouse pups from germline transmission-failed knockout chimeras. Transgenic Res..

