# Supplementary material for: Generation and characterization of CD19-iCre mice as a tool for efficient and specific conditional gene targeting in B cells
Source: Sci Rep. 2021 Mar 9;11:5524. doi: 10.1038/s41598-021-84786-6 (PMC7943778; doi:10.1038/s41598-021-84786-6)
Supplement: Supplementary file 1 — Supplementary Information. [file 41598_2021_84786_MOESM1_ESM.pdf]

Generation and characterization of CD19-iCre mice as a tool for efficient and specific conditional gene targeting in B cells

Tomoharu Yasuda, Yuichi Saito, Chisato Ono, Kazuhiko Kawata, Akemi Baba, and Yoshihiro Baba

Expanded Data Supplementary File With Full Western Blot (Figure 1e)

MW marker used for all Western Blots:
Bio-Rad Precision Plus Protein All Blue Standards

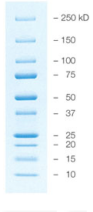

The first antibody was removed by the WB Stripping Solution (nacalai tesk). A second antigen-antibody reaction was conducted on the same blotting membrane. (IB: immunoblot)

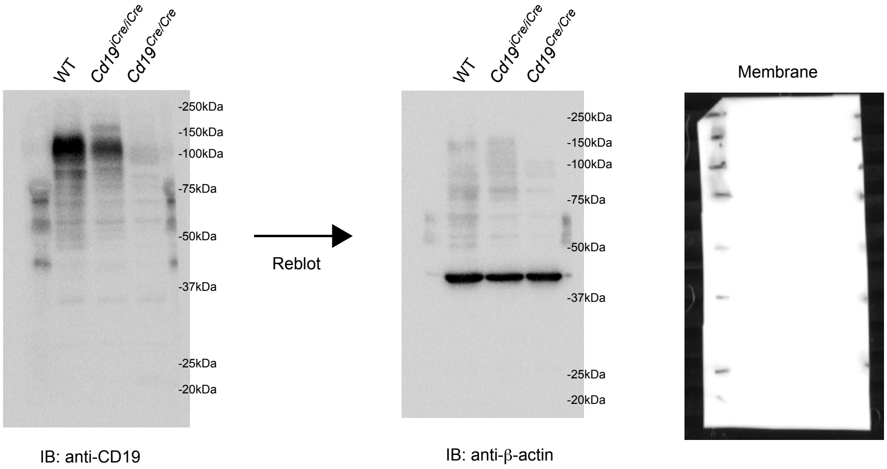

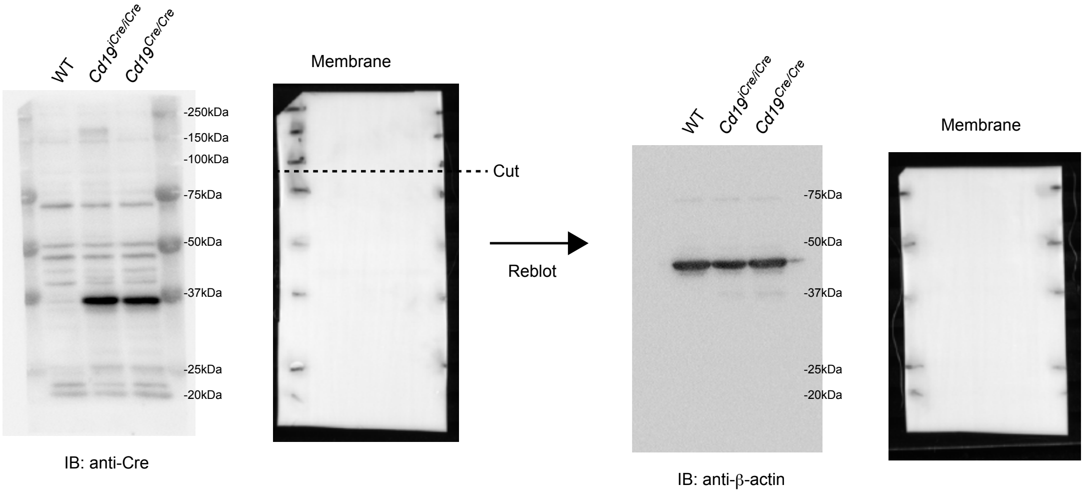

Original blots were cut for hybridization with anti-β-actin antibody.

Original gel images

Framed area indicated the cropped image as seen in the **Figure 1b** and **Figure 4a**.

MW marker: 100bp DNA ladder marker RTU (GeneDireX, BC-DM001-R500)

MW marker: 1kbp DNA ladder marker RTU (GeneDireX, BC-DM010-R500)

Figure 1b

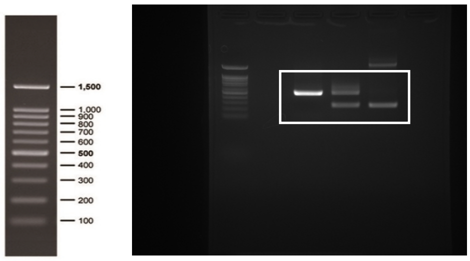

100bp DNA ladder marker

Figure 4a

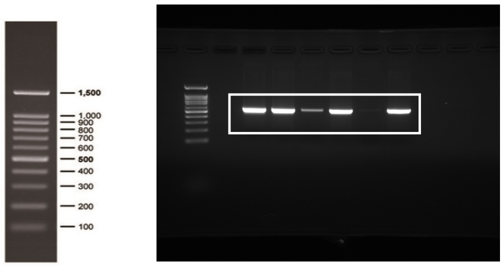

100bp DNA ladder marker

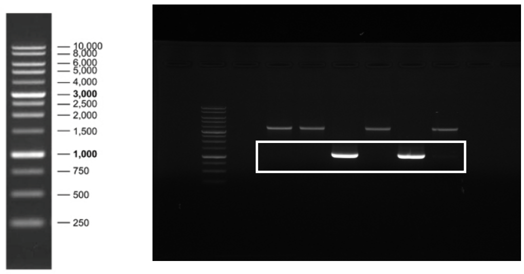

1kp DNA ladder marker
